# Experimental Investigation of Pore Pressure on Sandy Seabed around Submarine Pipeline under Irregular Wave Loading

**DOI:** 10.3390/s24020704

**Published:** 2024-01-22

**Authors:** Changjing Fu, Jinguo Wang, Tianlong Zhao

**Affiliations:** 1 National Engineering Research Center for Inland Waterway Regulation, Chongqing Jiaotong University, Chongqing 400074, China; cjfu@cqjtu.edu.cn; 2 Department of Civil Engineering, Monash University, Clayton, VIC 3800, Australia; 3 School of Earth Science and Engineering, Hohai University, Nanjing 210098, China; wang_jinguo@hhu.edu.cn

**Keywords:** wave flume tests, excess pore pressure, irregular waves

## Abstract

The propagation of shallow-water waves may cause liquefaction of the seabed, thereby reducing its support capacity for pipelines and potentially leading to pipeline settlement or deformation. To ensure the stability of buried pipelines, it is crucial to consider the excess pore pressure induced by irregular waves thoroughly. This paper presents the findings of an experimental study on excess pore pressure caused by irregular waves on a sandy seabed. A series of two-dimensional wave flume experiments investigated the excess pore pressure generated by irregular waves. Based on the experimental results, this study examined the influences of irregular wave characteristics and pipeline proximity on excess pore pressure. Using test data, the signal analysis method was employed to categorize different modes of excess pore-water pressure growth into two types and explore the mechanism underlying pore pressure development under the influence of irregular waves.

## 1. Introduction

The installation of subsea pipelines is a crucial task in the exploration and exploitation of offshore oil and gas resources [1]. As offshore oil exploration and exploitation continue to accelerate, subsea pipelines have become increasingly important for energy transportation. The primary installation methods for subsea pipelines are embedment (both deep and shallow) and direct placement. Deeply buried pipelines are expensive and challenging to construct, while directly placed pipelines are vulnerable to external loads. Therefore, shallow burial is the most commonly used method in pipeline construction. In general, marine pipeline projects are significantly affected by wave loads.

The wave pressure on the seabed surface undergoes periodical changes with wave propagation, and it will cause excess pore-water pressure while it transfers to the seabed. Thus, it changes the distribution of effective stress on the seabed. To ensure the normal operation of submarine pipelines and assess their stability under wave action, it is necessary to consider the impact of excess pore-water pressure on the seabed near pipelines, which is caused by the waves.

Wave-induced pore pressure has been the subject of numerous theoretical studies. Based on Biot’s consolidation theory [2], several researchers have proposed analytical solutions for wave-induced pore pressure on both finite and infinite seabeds [3,4]. Over time, more complex conditions have been investigated, including multi-layered seabeds [5], cross-anisotropic soil behavior [6], non-homogenous seabeds [7], fully dynamic soil behavior [8], combined wave and current loadings [9], and, more recently, the spatial variability of soil properties [10].

Various experimental studies have been conducted, in addition to theoretical studies, and these have been reported in the literature. Laboratory investigations primarily include one-dimensional compressive tests [11,12,13], two-dimensional wave flume tests [14,15,16], and centrifuge tests [17]. One-dimensional experiments have the advantage of providing numerous measuring points to monitor pore pressure in the vertical profile [18]. However, these tests typically focus on capturing the soil’s response to oscillatory pore pressure. Centrifuge tests can simulate the actual stress state of the seabed and reflect the spatial distribution of the seabed response [19], but their high cost limits their use. Furthermore, wave-making technology in centrifuges needs improvement to simulate more complex waves [20]. Two-dimensional wave flume tests can combine wave characteristics and investigate the distribution of pore-water pressure on the seabed in time and space [21,22]. Currently, wave flume tests are widely used in coastal engineering [23].

This study differs from previous research by considering the effects of irregular waves. This paper aims to present the results of two-dimensional wave flume tests conducted to gain a better understanding of the pore pressures induced by irregular waves on a seabed surrounding submarine pipelines. The study focused on the seabed pore pressure development and variation in the vicinity of the pipeline under the influence of irregular waves. The study also analyzed the distribution pattern of pore-water pressure around the seabed in the proximity of the pipeline. Lastly, the paper discussed the impact of wave parameters (relative water depth and relative wave height) and soil positions (vertical depth and horizontal distance) on irregular wave-induced pore pressure.

## 2. Laboratory Experiment

### 2.1. Experimental Facility

The prototype used in this experiment is an oil field in a very shallow sea area of China. The experiments were conducted in the Test Hall for Basic Sediment Law, located at the Nanjing Hydraulic Research Institute. The facility is equipped with a wave flume, a wave generator system, a flow field simulation system, and a wind simulation system. The wave flume in the laboratory measures 175 m in length, 1.2 m in width, and 1.5 m in depth. The wave period can range from 0.5 to 6 s. The wave paddle is operated using a horizontal sliding mechanism, and the guide rail is of the straight-line rolling type, with a maximum stroke of 600 mm. The velocity of the wave paddle was kept below 0.75 m/s.

**Figure 1:**
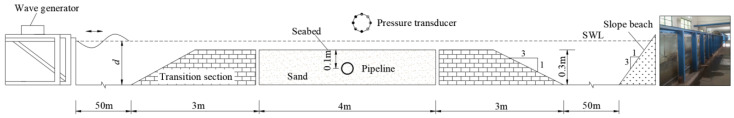
A schematic diagram of the test section.

The experimental segment, located in the center of the wave flume, was designed to study the interaction between waves, soil, and pipelines. It includes a buried pipeline within the sand and spans 4 m in length, 0.6 m in width, and 0.3 m in depth. The test segment is bordered by transition sections made of cement mortar, each measuring 3 m in length, 0.6 m in width, and 0.3 m in height. The transition sections are inclined at a ratio of 3H:1V. A representative cross-section is shown in Figure 1.

As shown in the figure above, the static water depth is defined as *d*. An absorbing slope was set up at the end of the flume. Waves generated by the wave generator, positioned at one end of the flume, propagated through the test segment and were blocked by the opposing sloping beach, graded at 1H:3V. The length of the flume used in this experiment was 175 m, and the distance between the absorbing slope and the test section was 50 m. At the absorbing slope, a permeable biomimetic lawn carpet was used to lay in the air and eliminate the reflected energy of the incident wave. Through calculation, after energy dissipation by flexible materials, the energy of the reflected wave at the absorbing slope position was only 20% of that of the incident wave, and the energy of the reflected wave in the experimental section was only 2% of that of the incident wave. To simplify the research and focus on the influence of relative water depth and relative wave height on the distribution of pore-water pressure around the pipe, the authors ignore the influence of reflected waves in this article, and do not separate the reflected and the incident components of waves.

Before the experiment, the sand bed in the transition section needed to be constructed first. After the sand bed in the transition section was consolidated, the sand bed in the test section had to be filled. The sand bed in the test section was constructed in layers with a height of 5 cm by controlling the dry density to 1.59 g/cm^3^. After each layer was consolidated, the next layer of the sand bed had to be filled. After the filling of the third layer of the sand bed was completed, pipeline laying was carried out, and then, subsequent sand bed layers were constructed.

**Figure 2:**
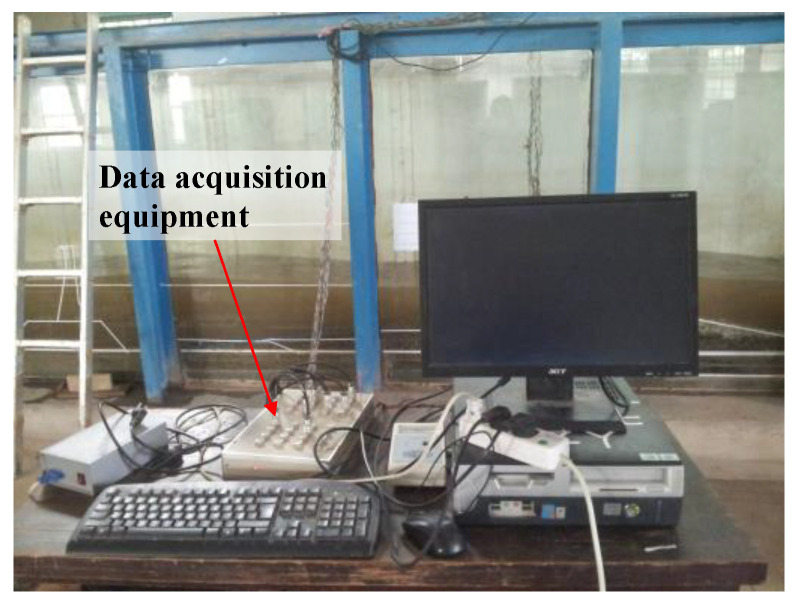
Data acquisition and processing system.

For this experiment, a point pressure sensor that operates underwater, developed by the Tianjin Institute of Water Transportation Engineering, was utilized. Three wave height meters were set up in this experiment, which were placed upstream, midstream, and downstream of the test section, respectively. Additionally, 2000-type data acquisition equipment, which has the capability of acquiring 64-channel data simultaneously, was used. This equipment was developed by the Tianjin Research Institute for Water Transport Engineering of China to ensure that the pressure sensors were acquired synchronously, as shown in Figure 2.

**Figure 3:**
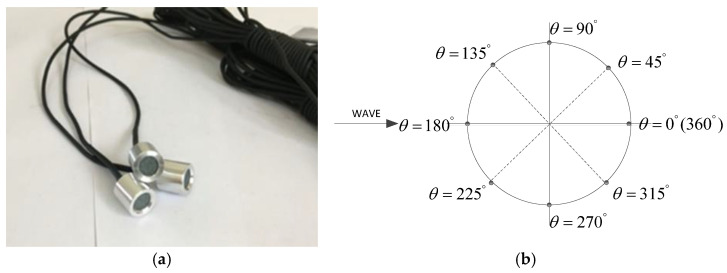
Pore-water pressure sensor: (**a**) sensors; (**b**) distribution.

**Figure 4:**
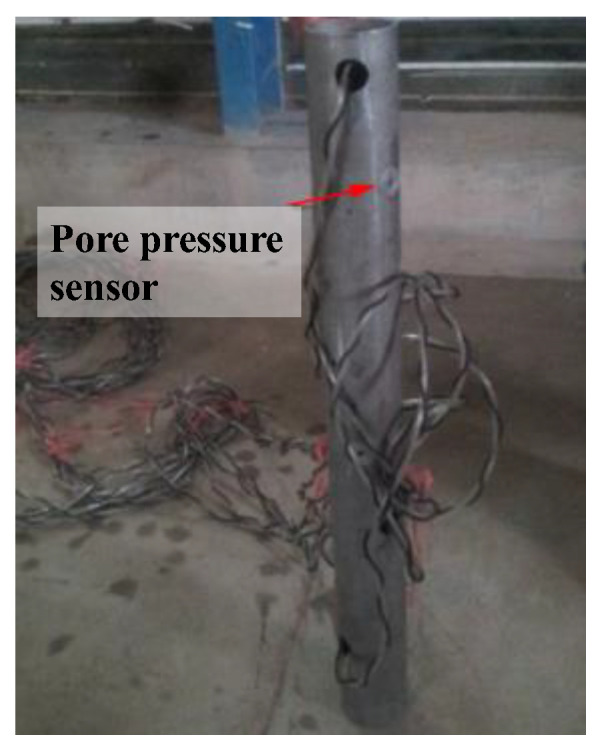
Test pipeline.

A stainless-steel pipeline with a diameter of 0.06 m and a length of 0.6 m was tested. The cylindrical surface of the pipeline was equipped with eight pressure transducers. These pressure sensors, which were model BWK, as depicted in Figure 3, were mounted in pairs around the octant of the cylinder, as illustrated in Figure 4.

Achieving both wave and sediment similarity conditions was a challenging task due to the complex dynamic process of the seabed response under wave action. This study chose to follow the gravity similarity law for selecting the appropriate scale. Table 1 presents the similarity scales of common physical quantities. The test model scale used was 1/20.

### 2.2. Experimental Soil Property Parameter

For the selection of experimental sand, some scholars [20,24,25] have directly conducted model experiments using sand taken from on-site sea or riverbed areas. Due to limitations in experimental conditions, sampling of the seabed in the sea area was not used in this study. The soil samples utilized in this study were river sand, manually screened for consistency. Due to constraints imposed by flume test conditions, the soil samples were not scaled down to match the test setup. The sand particles were uniform, with an average particle size of approximately 2 mm. The soil properties are detailed in Table 2. To determine the soil permeability (*k_s_*), a constant-head permeability test was conducted, while the shear modulus (*G_s_*) was assessed through an odometer test. Based on the results of the odometer test, the elastic modulus (*E_s_*) of the fine sand was derived. Subsequently, by employing Poisson’s ratio relationships, the shear modulus (*G_s_*) of the sand could be calculated.

### 2.3. Experimental Wave Parameter

**Figure 5:**
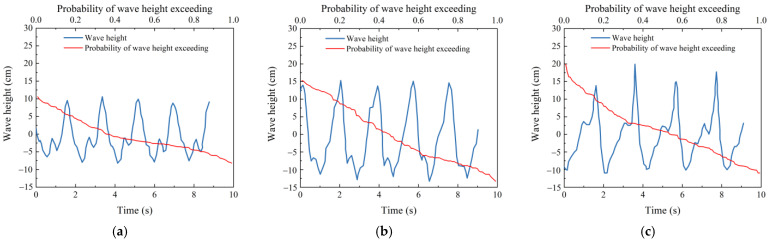
Pattern of irregular waves in the test. (**a**) *H*_1/3_ = 0.1 m. (**b**) *H*_1/3_ = 0.15 m. (**c**) *H*_1/3_ = 0.18 m.

In this test model, the seabed thickness was set to 0.3 m, and irregular wave loading was applied. According to the measurement results of the wave height meter, the wave height *H* ranged from 11 to 20 cm, with the waveform illustrated in Figure 5. The period *T* fell between 1.92 and 1.97 s. The static water depth was observed and measured by a ruler on the side of the flume, varying between 15 cm, 25 cm, 40 cm, and 50 cm, respectively. The test water temperature was maintained at 26 °C, and the dynamic viscosity coefficient was *υ* = 8.77 × 10^−7^ m^2^/s.

**Figure 6:**
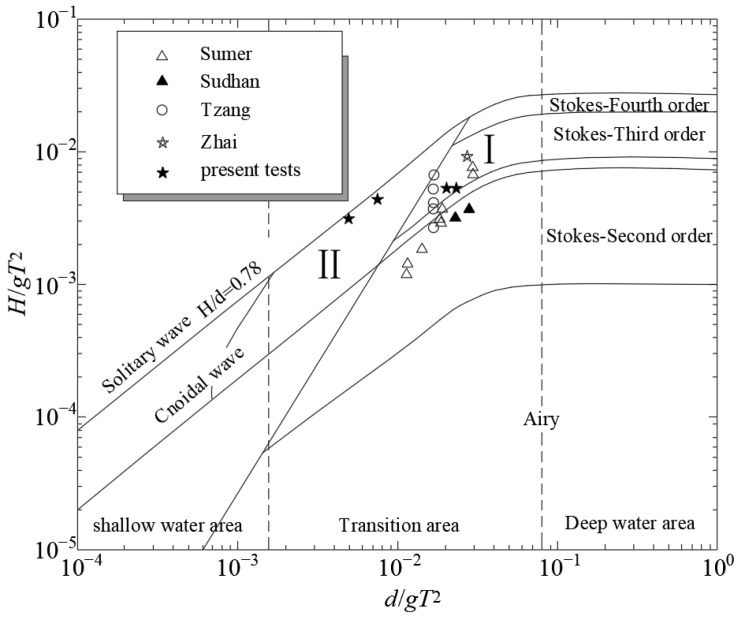
The wave conditions of this study [15,17,19,20]. In the figure, region I is suitable for Stokes third-order wave theory and is suitable for deep water; Region II is suitable for the theory of elliptical cosine waves and can be applied to shallow water.

The primary objective of the experiments was to examine the dynamic response traits of excess pore-water pressure on the sandy seabed surrounding the pipeline under the influence of irregular wave loads. A comparison of wave conditions between this test and previous studies is presented in Figure 6. As illustrated in Figure 6, the wave conditions of this test were categorized into two wave theory regions, specifically, regions I and II. During the experiment, the authors found that by about 50 *T*_1/3_, the measured pore pressure data had stabilized within a specific range. To further ensure the stability of the results, the authors conducted 100 *T*_1/3_ of testing on each group of experiments.

### 2.4. Experimental Process

The experimental process involved the following steps:

1. Before conducting the wave tests, corresponding spectrum tests were performed to obtain the anticipated control signals for the wave generator. Based on the collected wave spectrum, the wave maker was calibrated. According to statistical data and spectral shape, the wave series was determined.
2. To ensure accurate results, it was crucial to control the saturation of soil samples when studying the seabed. Since the seabed was already assumed to be saturated, proper sample preparation was necessary. We used the underwater sand loading method to prepare the samples, which guaranteed full saturation.
3. To install soil samples in the seabed area of the test section, we followed the steps given below. First, we opened the inlet valve and adjusted it until the water level was slightly below the surface of the model seabed. Next, we installed the pre-soaked soil samples. After that, we placed the pipeline on top of the sand samples at a predefined distance of approximately 10 cm from the seabed surface. We filled the samples continuously until the height was slightly above the top surface of the model seabed, and then, smoothed the bed surface. We let the samples consolidate for 24 h under the influence of self-weight, and added water continuously to the predetermined depth.
4. Before measurement, all air within the pressure sensors was purged to ensure test accuracy. The pressure sensors were then connected to a computer to verify proper functioning. Before starting the wave-making process, the pore pressure sensor data was reset to zero under still water conditions after it stabilized.
5. During the spectrum collection process, control signals were obtained for the wave generator, which were then used to conduct wave tests.
6. The values of excess pore-water pressure were collected every 0.02 s, with a total acquisition time of 200 s.
7. The test conditions (as shown in Table 3) were restored to their original settings, and the next set of tests was performed.

## 3. Experimental Results and Discussions

### 3.1. Excess Pore Pressure Distribution around Pipeline

**Figure 7:**
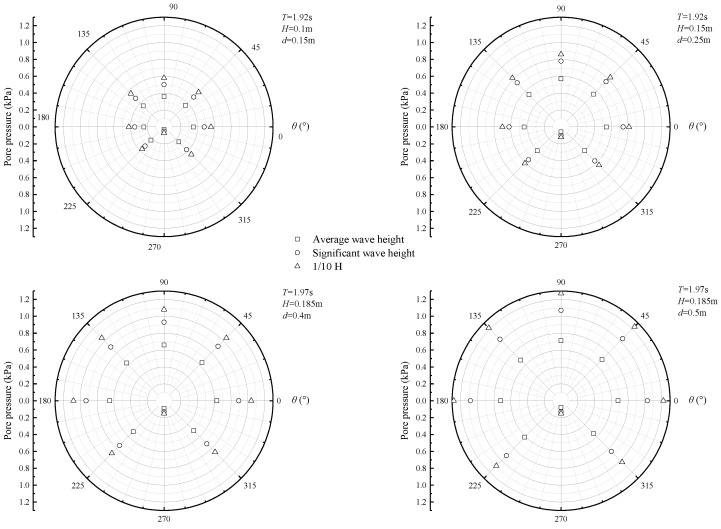
The spatial patterns of irregular wave-induced excess pore pressure on the seabed surrounding the pipeline.

The waves propagating on the surface of the ocean can cause dynamic pressure fluctuations on the seabed. These fluctuations, in turn, caused an increase in pore pressure, ultimately leading to variations in stresses on the seabed. In Figure 7, the maximum amplitudes of irregular wave-induced excess pore pressure around the pipeline are illustrated. The largest excess pore pressure amplitude was observed at the top of the cylinder, while the smallest was found at the bottom. This phenomenon becomes more pronounced as the water depth increases.

**Figure 8:**
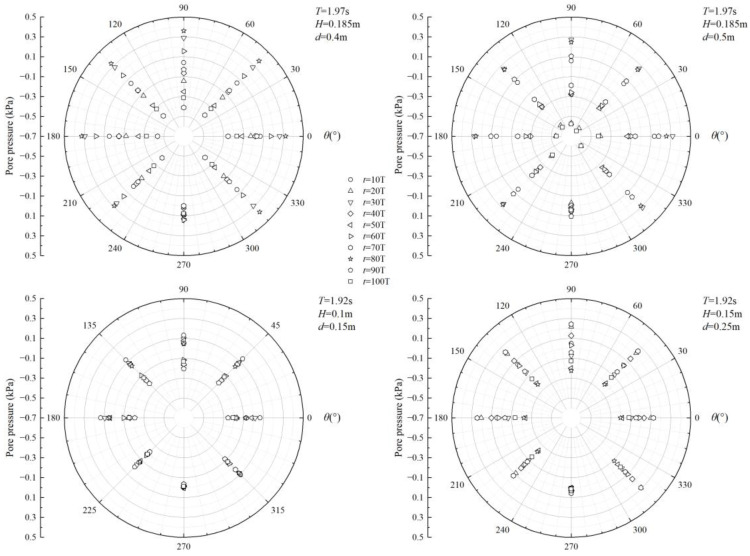
The spatial patterns of excess pore pressure caused by irregular waves at different times vary.

Figure 8 illustrates the changes in the seabed’s response to excess pore pressure amplitude around the pipeline due to irregular waves. As shown in the figure, the amplitude of excess pore pressure oscillates. Significant changes were observed in excess pore pressure amplitude at all positions along the pipeline, except for the bottom. The figure also reveals that when *H*/*d* was large, the variation in the seabed’s response to excess pore pressure amplitude around the pipeline was minimal. This suggests that wave parameters significantly impact excess pore pressure in the pipeline’s surroundings.

**Figure 9:**
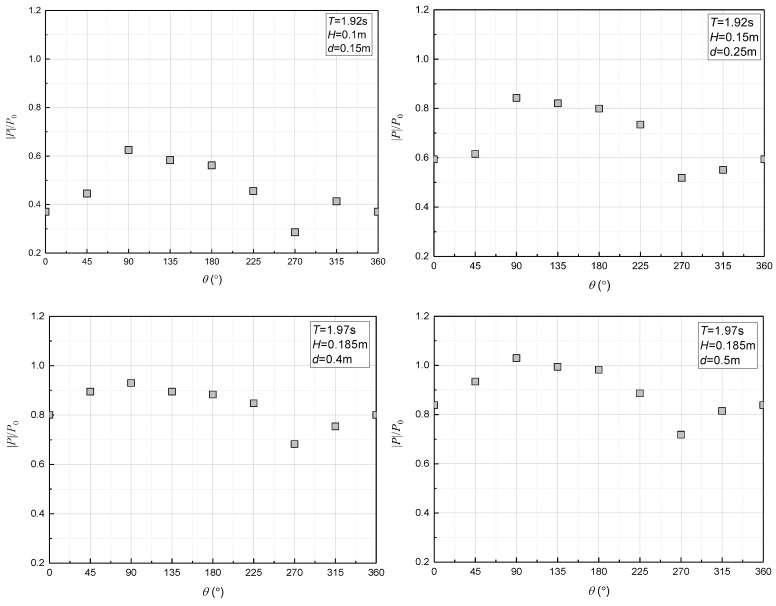
The distributions of the irregular wave-induced excess pore pressure (|*P*|/*P*_0_) around the pipeline.

The graph in Figure 9 shows how the maximum amplitudes of excess pore pressure (|*P*|/*P*_0_) caused by irregular waves were distributed around the pipeline. It is clear that the excess pore pressure near the pipeline follows a sinusoidal pattern, with the highest amplitude being at the top of the cylinder and the lowest at the bottom. Moreover, as the water depth increases, this pattern becomes more accentuated.

### 3.2. Type Analysis of Excess Pore Pressure Response around the Pipeline

The experiments conducted in the laboratory and field measurements showed two mechanisms for the wave-induced seabed response that led to a rise in excess pore pressure. The first mechanism occurs due to the excess pore pressure’s residual or progressive nature. In contrast, the second one is generated by transient or oscillatory excess pore pressure accompanied by the damping of amplitude and phase lag in the excess pore pressure. Signal analysis can effectively extract the features of these two kinds of excess pore pressure signals [26].

The wave response signal of the seabed is a non-stationary signal that requires time and frequency analysis. To analyze time-varying or transient signals, it is necessary to combine time and frequency parameters. Wavelet analysis is a new signal processing technique that has excellent time–frequency localization characteristics. In this paper, we used the Daubechies wavelet to analyze the response signal of the excess pore-water pressure of the seabed under wave load.

**Figure 10:**
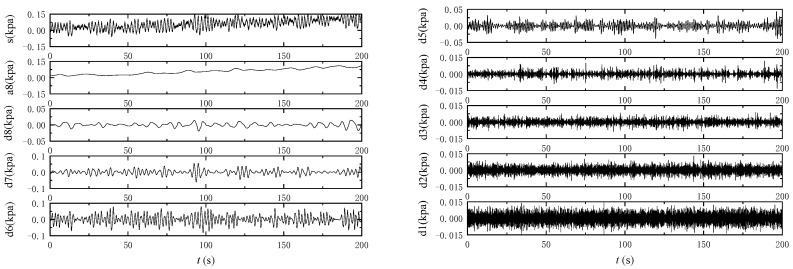
Decomposition chart of measured excess pore pressure wavelet on sandy seabed.

As illustrated in Figure 10, the signal was decomposed into eight layers. The original signal *s*, which is the actual measured excess pore-water pressure, is expressed as the sum of its components:

*s* = *a*8 + *d*8 + *d*7 + *d*6 + *d*5 + *d*4 + *d*3 + *d*2 + *d*1 (1)

in which *a*8 is the low-frequency component of the eighth layer after decomposition, and *d_j_* is the high-frequency signal on level *j*.

From Figure 10, it is evident that the wavelet component *a*8 mirrors the cumulative residual component of excess pore-water pressure, while the wavelet component *d*5 reflects the instantaneous oscillation component of the same.

**Figure 11:**
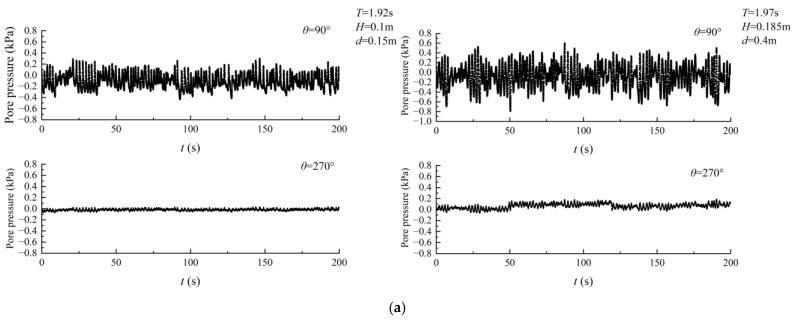

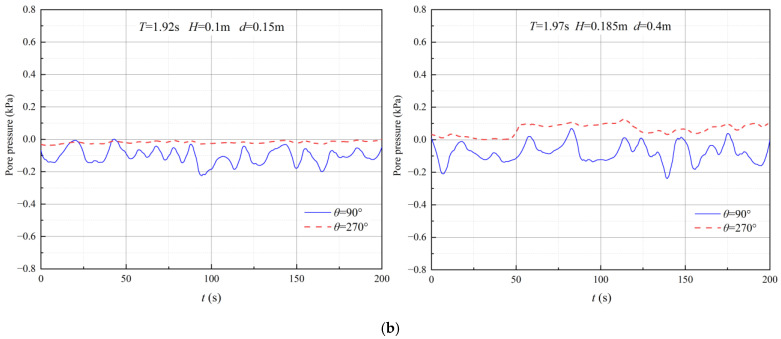
First type of response of excess pore pressure. (**a**) Excess pore pressure curve with loading time. (**b**) Cumulative excess pore-water pressure.

The seabed surrounding the pipeline responded differently to excess pore pressure under wave action, which can be categorized into two types. The first type is shown in Figure 11a. During the initial stage of irregular wave loading, the excess pore pressure on the seabed at both the top and bottom of the pipeline decreased. However, as the loading process continued, the excess pore-water pressure at the bottom of the pipeline accumulated. Although there was partial dissipation of excess pore-water pressure at the bottom of the pipeline during the entire loading process, the overall trend was that the pressure accumulated. At the top of the pipeline, the seabed exhibited oscillatory behavior in the amplitude of excess pore-water pressure.

The figure shown in Figure 11b displays the low-frequency component of the seabed excess pore pressure response signal obtained through wavelet analysis using the Daubechies wavelet. The figure indicates the process of pore pressure accumulation and dissipation at the bottom of the pipeline, which becomes more pronounced as the wave height increases.

Compared with the fluctuation of excess pore pressure under the action of regular waves provided by Wang [27], the excess pore pressure induced by irregular waves exhibits significant fluctuations, with noticeable irregularity in its amplitude. This variation is markedly distinct from the changes in excess pore pressure observed under the influence of regular waves on the sandy seabed.

**Figure 12:**
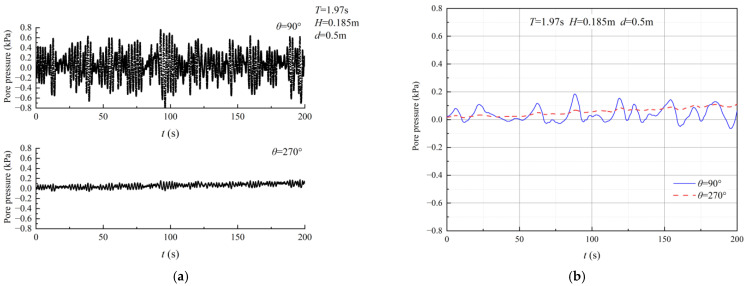
Second type of response of excess pore pressure. (**a**) Excess pore pressure curve with loading time. (**b**) Cumulative excess pore-water pressure.

Figure 12a demonstrates the second type of behavior. In contrast to the first type, excess pore pressure at both the top and bottom of the pipe increased during the initial loading phase of the irregular wave. However, as the loading continued, there was partial dissipation of the excess pore-water pressure at the bottom of the pipeline, but there was still a tendency for it to accumulate throughout the entire loading process.

Based on the Daubechies wavelet, the response signal of seabed excess pore pressure was wavelet-analyzed, and Figure 12b shows the low-frequency component. The process of pore pressure accumulation and dissipation at the bottom of the pipeline is evident in the figure, with the phenomenon of pore pressure accumulation becoming more pronounced as the depth of the water increases.

### 3.3. Effect of Relative Water Depth and Relative Wave Height

**Figure 13:**
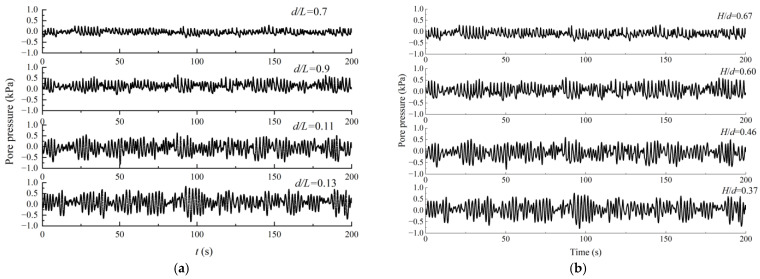
Excess pore pressure for various conditions. (**a**) Relative water depth *d*/*L* (*θ* = 45°). (**b**) Relative wave height *H*/*d* (*θ* = 135°).

The importance of relative water depth, *d*/*L*, in analyzing wave-induced seabed responses has been widely recognized. Figure 13a shows the excess pore pressure measurements from sensor P2 (*θ* = 45°) for various relative water depths (*d*/*L* = 0.7, 0.9, 0.11, 0.13). As demonstrated in the figure, there was a noticeable increase in excess pore pressure as the relative water depth *d*/*L* increased. This indicates that the relative water depth *d*/*L* significantly impacts the excess pore pressure. Sensor P4 (*θ* = 135°) recorded the variation in excess pore pressure for different relative wave heights (*H*/*d*= 0.67, 0.60, 0.46, 0.37), which is illustrated in Figure 13b. The figure clearly shows that as the relative wave height *H*/*d* decreased, there was a significant increase in excess pore pressure.

### 3.4. Effect of Vertical Depths and Horizontal Distances

**Figure 14:**
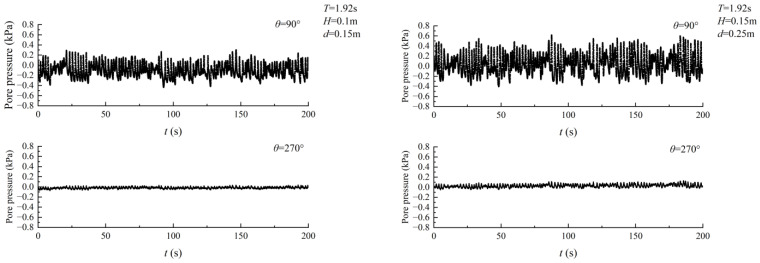

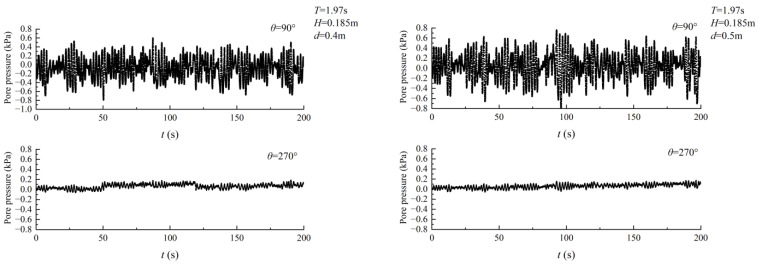
Pore pressure changes at different depths.

The pore pressure changes at different depths are shown in Figure 14. It can be found that under the same wave conditions, the pore-water pressure increases gradually with the increase in water depth; that on the upper half of the seabed caused by waves greatly fluctuates, and the amplitude on the lower half remains fundamentally stable.

**Figure 15:**
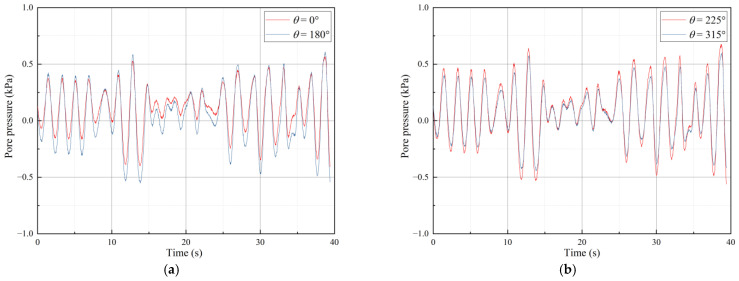
Pore pressure for different horizontal distances. (**a**) Δ*x* = 0.06 m. (**b**) Δ*x* = 0.04 m.

Figure 15 presents the pore pressure for different horizontal distances, and the horizontal distance is the distance between two measuring points (sensors) located on the same horizontal plane on the pipe. It can be found that under the same wave conditions, the oscillatory amplitudes of pore-water pressure increase with the increase in the horizontal distance, which may be caused by the energy dissipation in the process of wave propagation.

## 4. Conclusions

The primary aim of this study was to explore the excess pore pressure induced by irregular waves. To achieve this objective, a series of two-dimensional wave flume experiments were conducted. Based on the presented experimental results, the following conclusions can be drawn:

1. The excess pore pressure induced by irregular waves exhibited significant fluctuations, with noticeable irregularity in its amplitude. This variation was markedly distinct from the changes in excess pore pressure observed under the influence of regular waves on the sandy seabed.
2. The response of excess pore-water pressure induced by irregular waves around the pipeline was primarily oscillatory. Nonetheless, in addition to the oscillatory response, there was also a phenomenon of excess pore pressure accumulation at the bottom of the pipeline.
3. The excess pore pressure generated by irregular waves surrounding the pipeline exhibited a sinusoidal distribution. After analyzing the excess pore pressure data measured at eight measuring points, it was found that the greatest dynamic excess pore pressure was recorded at the cylinder’s apex, with the lowest pressure detected at the base.
4. The excess pore pressure was significantly affected by the relative water depth (*d*/L) and the relative wave height (*H*/*d*). As the relative water depth (*d*/*L*) increased, the excess pore pressure increased noticeably. Similarly, as the relative wave height (*H*/*d*) decreased, the excess pore pressure also increased considerably.
5. The excess pore-water pressure gradually increased with the increase in water depth under the same wave height (*H*) and period (*T*). In addition, this effect was the result of wave–seabed–structure coupling. The fluctuation of the excess pore pressure around the pipeline was significantly influenced by the permeability of the seabed soil, but it was also influenced by its own location. On the upper half of the seabed, the pressure fluctuations caused by waves were significant, while on the lower half, the amplitude remained relatively stable.
6. Previous studies have generally assumed that a sandy seabed is less prone to the accumulation of excess pore pressure. However, in this experiment, it was found that the accumulation of excess pore pressure around the pipeline, especially at the bottom, may result in the instability of the pipeline. Therefore, in practical engineering, the potential instability of pipelines caused by the accumulation of excess pore pressure in seabed soil needs to be taken into consideration.
7. As a limitation of this study, the authors overlooked the influence of reflected waves on the experimental results. In the experiments, the efficiency of the absorbing slope had a significant impact on the test results. In future research, the author will further slow down the slope ratio of the absorbing slope at the end of the flume to further reduce the impact of reflected waves and their accumulation on the experimental results to improve their accuracy.

## Figures and Tables

**Table 1:** Similarity criterion of a common physical quantity in wave flume test.

Index	Length	Wave Height	Velocity	Time	Pressure
Similarity ratio	1/20	1/20	1/4.47	1/4.47	1/20

**Table 2:** The soil properties.

Properties	Shear Modulus *G_s_*	Poisson’s Ratio *υ_s_*	Porosity *n_s_*	Permeability *k_s_*	Saturation *S_r_*
Values	4 × 10^6^ Pa	0.3	0.40	7.5 × 10^−4^ m/s	1

**Table 3:** The test conditions.

Conditions	*H*_1/3_ (cm)	*d* (cm)	*T*_1/3_ (s)	Test Duration
1	10	15	1.92	100 *T*_1/3_
2	15	25	1.92	100 *T*_1/3_
3	18	40	1.96	100 *T*_1/3_
4	18	50	1.96	100 *T*_1/3_

## Data Availability

The data that support the findings of this study are available from the corresponding author upon reasonable request.
